# Supplementary material for: WordCommentsAnalyzer: A windows software tool for qualitative research
Source: F1000Res. 2018 Sep 4;7:536. Originally published 2018 May 3. [Version 2] doi: 10.12688/f1000research.14819.2 (PMC6137411; doi:10.12688/f1000research.14819.2)
Supplement: Supplementary file 3 [file f1000research-7-17614-s0002.tgz › b2a79496-4ada-47c4-9682-a99ce867836d.zip!/Eight Iranian Health Journals/Journal of Knowledge and Health_2.docx]

© Journal of Knowledge and Health. This work is licensed under CC BY 4.0 (https://creativecommons.org/licenses/by/4.0/).

Volume & Issue: دوره 12، شماره 1396:4

Title: کیفیت خواب خودگزارشی و عوامل مرتبط با آن در بالغین – یک مطالعه مبتنی‌بر جمعیت

http://knh.shmu.ac.ir/index.php/site/article/view/1783

مقدمه: کمیت و کیفیت خواب انسان با سلامتی او در ارتباط است. هدف این مطالعه بررسی عوامل دموگرافیک مرتبط با کیفیت ذهنی خواب خودگزارشی در جمعیت بالغین شهر شاهرود است. مواد و روش‌ها: در فاز دوم مطالعه چشم پزشکی شاهرود، تعداد 4737 نفر (دامنه سنی 45 تا 69 سال) از شرکت‌کنندگان فاز اول مجدد در سال 2014 مورد سنجش و پرسشگری قرار گرفتند. در این مطالعه متغیرهای سن، جنس، وضعیت تأهل، وضعیت اقتصادی (با سه سطح ضعیف، متوسط و بالا)، تحصیلات و مصرف دخانیات و ارزیابی ذهنی کیفیت خواب اندازه‌گیری شد. کیفیت ذهنی خواب با استفاده از این سؤال که "در طی ماه گذشته در کل، کیفیت خواب خود را چگونه توصیف می‌کنید؟" مورد ارزیابی قرار گرفت. رابطه عوامل خطر با کیفیت خواب با استفاده از مدل رگرسیون لجستیک بررسی شد. نتایج: در این مطالعه 5/23% زنان در مقایسه با 8/10% مردان و در کل جمعیت 3/18% (862 نفر) کیفیت خواب خود را بد و خیلی بد توصیف کرده‌اند. زن بودن (نسبت شانس=5/2 (1/2-1/3))، وضعیت اقتصادی متوسط (نسبت شانس=2/1 (0/1- 5/1)) و مصرف دخانیات (نسبت شانس=5/1 (1/1- 9/1)) از عوامل خطر مرتبط با کیفیت بد خواب بودند. نتیجه‌گیری: نتایج مطالعه حاضر نشان داد که کیفیت خواب زنان نسبت‌به مردان پایین‌تر است و مصرف سیگار هم به‌عنوان یک عامل خطر در کاهش کیفیت خواب مؤثر است. لذا توجه به نقش عوامل محیطی برای بهبود کیفیت خواب می‌تواند مؤثر باشد.

Title: رده‌بندي وضعيت عوامل خطر بيماري‌هاي غيرواگير در استان‌هاي مختلف ايران با استفاده از روش‌هاي چند متغيره عاملي

http://knh.shmu.ac.ir/index.php/site/article/view/1808

مقدمه: بيماري‌هاي غيرواگير به متغیرهای خطرزا مثل مصرف دخانيات، تغذيه نامناسب و نبود تحرک بدني وابسته هستند. هدف از اين مطالعه، به‌کارگيري روش عاملي در استخراج شاخص تلفيق يافته‌اي از متغیرهای خطرزا و رتبه‌بندي استان‌ها براساس آن مي‌باشد. مواد و روش‌ها: داده‌هاي اين مطالعه، شامل 20 متغیر خطرزا به تفکيک استان و جنسيت در افراد گروه سني 15 تا 64 سال، برگرفته از گزارش سازمان نظارت عوامل خطر بيماري‌هاي غيرواگير در ايران در سال 1388 مي‌باشد. تحلیل عاملي، با به‌کارگيري روش مؤلفه‌هاي اصلي، جداگانه براي مردان و زنان انجام گرفت. در نهايت با محاسبه امتيازات عاملي براساس عامل اول، استان‌ها رتبه‌بندي شدند. نتايج: براساس عامل اول، براي مردان، به‌ترتيب استان‌هاي آذربايجان شرقي، قم، اردبيل و مازندران بيشترين خطر ابتلا به بيماري‌هاي غيرواگير و استان‌هاي سيستان و بلوچستان، خراسان جنوبي، کرمان و هرمزگان کمترين خطر را داشتند. در زنان، به‌ترتيب استان‌هاي مازندران، قم، کرمانشاه و آذربايجان شرقي بيشترين خطر و استان‌هاي سيستان و بلوچستان، خراسان جنوبي، چهارمحال و بختياري و هرمزگان کمترين خطر را داشتند. نتیجه‌گیری: نتايج حاصل از اين مطالعه نشان داد كه تجزیه و تحلیل عاملي به‌خوبي مي‌تواند سيماي خطر ابتلا به بيماري‌هاي غيرواگير را در استان‌هاي مختلف ايران نشان دهد. همچنین براساس نتايج مطالعه حاضر استان‌هايي که خطر بالاتري در ابتلا به بيماري‌هاي غيرواگير دارند نيازمند توجه بيشتر مسئولين جهت ارایه آموزش‌هاي تغذيه‌اي و بهداشتي هستند.

Title: ارزیابی پراکنش گرد و غبار با استفاده از مدل پلوم گوس: مطالعه موردی کارخانه سیمان دورود

http://knh.shmu.ac.ir/index.php/site/article/view/1748

مقدمه: پخش گرد و غبار به‌عنوان مهمترین آلودگی ناشی از تولید سیمان شناخته می‌شود. در این مطالعه پراکنش گرد و غبار (PM10) حاصل از دودکش کارخانه سیمان دورود با استفاده از مدل گوس در شرایط مختلف جوی بررسی می‌گردد. مواد و روش‌ها: در این مطالعه، دستگاه Westech Instrument برای سنجش ذرات خروجی براساس استاندارد ISO-9096 به‌روش گراویمتریک به‌کار برده شد. مدل پلوم گوس برای تخمین پراکنش PM10 منتشره از دودکش کارخانه سیمان در فواصل زمانی مشخص مورد استفاده قرار گرفت. نتایج حاصله با استاندارد 24 ساعته سازمان حفاظت محیط زیست آمریکا (EPA) مقایسه شد. اثر شاخص‌های هواشناسی شامل دما، سرعت باد و رطوبت نسبی نیز روی پراکنش گرد و غبار مورد بررسی قرار گرفت. نتایج: نتایج حاصل از پژوهش حاضر نشان داد که حداکثر و حداقل غلظت پیش‌بینی شده در هوای آزاد در فاصله 1800 و 500 متری در شرایط پایدار و کمی پایدار با مقدارµg/m³12/118 و µg/m³13/53 از منبع انتشار به‌دست آمد. کاهش سرعت باد و همچنین افزایش پایداری هوا نقش مهمی در افزایش غلظت ذرات گرد و غبار در فضای اتمسفر اطراف کارخانه سیمان داشته است.نتیجه‌گیری: مقایسه نتایج حاصل از این مطالعه با استاندارد EPA نشان داد که در هیچ دوره نمونه‌برداری غلظت PM10 از استاندارد بالاتر نبوده است. بررسی‌های میدانی بیشتر به‌صورت سنجش PM10 در بالادست و پایین دست کارخانه سیمان و مقایسه نتایج با یافته‌های حاصل از مدل ضروری می‌باشد.

Title: بررسی مقایسه‌ای یافته‌های سونوگرافی و آسپيراسيون سوزني ظریف در تشخیص گره‌های تیروئیدي بیماران مراجعه‌کننده به مراکز درمانی شهر قزوین

http://knh.shmu.ac.ir/index.php/site/article/view/1726

مقدمه: به‌علت شیوع فراوان و احتمال وجود بدخیمی، تشخیص دقیق گره‌های تیروئیدی اهمیت زیادی دارد. هدف این مطالعه بررسی میزان دقت سونوگرافي و آسپيراسيون سوزني ظریف در تشخیص ندول‌هاي تيروئيدی بود. مواد و روش‌ها: در این مطالعه مقطعی که بر روی 80 بیمار مبتلا به گره‌های تیروئیدی مراجعه‌کننده به مراکز درمانی شهر قزوین طي سال 1394 انجام شد. اطلاعات بالینی و نتایج بررسی سونوگرافی، سیتوپاتولوژی و هیستوپاتولوژی گره‌ها ثبت گردید. از نرم‌افزار آماري SPSS و ضریب توافق کاپا جهت تجزيه و تحليل داده‌ها استفاده شد. همچنین به کمک نمودار راک سطح زير منحني و فاصله اطمينان داده‌ها مشخص گردید. نتایج: در سونوگرافی 80 بیمار به‌ترتیب 5/52% دارای ضایعات خوش‌خیم و 5/37% دارای ضایعات بدخیم بودند. حساسیت، ویژگی، ارزش اخباری مثبت، ارزش اخباری منفی به‌ترتیب 100، 100، 65 و 6/89 و دقت 25/91 درصد بود. در آسپیراسیون سوزنی ظریف، 2 مورد غیرقابل تشخیص، 47 مورد خوش‌خیم ،3 مورد آتیپی با اهمیت نامشخص، 7 مورد نئوپلاسم فولیکولی و یا مشکوک به آن، 6 مورد مشکوک به بدخیمی و 15 مورد بدخیمی بودند. حساسیت، ویژگی، ارزش اخباری مثبت، ارزش اخباری منفی، به‌ترتیب 100، 87، 95 و 100 و دقت 25/96 درصد بود. نتیجه‌گیری: آسپيراسيون سوزني ظریف، نسبت‌به سونوگرافی از دقت بیشتری برخوردار بوده و روش مقرون به صرفه‌تری در تشخیص گره‌های‌ تیروئید محسوب می‌شود.

Title: بررسی تأثیر همودیالیز با مایع دیالیز سرد بر کاهش میزان خارش اورمیک در بیماران همودیالیزی

http://knh.shmu.ac.ir/index.php/site/article/view/1802

مقدمه: خارش اورمیک یکی از شایع‌ترین و ناتوان‌کننده‌ترین عوارض بیماران همودیالیزی بوده که 50 تا 90 درصد آنها را گرفتار می‌کند. خارش می‌تواند منجر به عفونت پوستی، پوسته پوسته شدن، اضطراب، اختلالات خواب، اختلال در عملکرد اجتماعی و... در بیمار گردد. هدف از این مطالعه بررسی تأثیر همودیالیز با مایع دیالیز سرد بر کاهش میزان خارش اورمیک در بیماران همودیالیزی بود. مواد و روش‌ها: این مطالعه از نوع کارآزمایی بالینی بصورت قبل و بعد و دو سو کور بوده که تابستان 1391 در بخش همودیالیز بیمارستان فاطمیه سمنان انجام شد. از بین همه بیماران بخش همودیالیز، 35 نفر واجد شرایط مطالعه شدند. برای جمع‌آوری داده‌ها از پرسشنامه اطلاعات دموگرافیک، پرونده پزشکی و مقیاس خارش استفاده گردید. بیماران سه جلسه در هفته، هر جلسه چهار ساعت به مدت یکماه با همودیالیز استاندارد (°c37) و ماه دوم با همان شرایط یکسان، با همودیالیز سرد (°c35) همودیالیز شدند. نتایج: نتایج نشان داد که میانگین و انحراف معیار خارش در مرحله استاندارد و سرد همودیالیز به‌ترتیب 3/10±0/23 و 3/1±8/8 بود. داده‌ها بیانگر این است که خارش بیماران در مرحله همودیالیز سرد به‌طور معنی‌داری کاهش پیدا کرده بود (001/0P<). اطلاعات به‌وسیله آزمون‌های آماری T زوجی و ویلکاکسون در سطح معنی‌داری 5% تجزیه و تحلیل شدند. نتیجه‌گیری: همودیالیز سرد می‌تواند باعث تسکین میزان خارش اورمیک در بیماران همودیالیزی بشود.

Title: تأثیر مصرف مکمل کراتین بر CK,LDH پس از یک فعالیت خسته‌کننده شدید

http://knh.shmu.ac.ir/index.php/site/article/view/1832

مقدمه: یکی از عوامل بروز خستگی در ورزشکاران، تجمع اسیدلاکتیک در خون بعد از انجام یک فعالیت سنگین می‌باشد، در هنگام خستگی برخی از آنزیم‌ها نظیر LDH و CK افزایش می‌یابد متعاقب آن آسیب‌های سلولی به عضلات فعال وارد می‌شود، این مسأله خصوصاً در ورزشكاراني که فعالیتی را در مدت زمان کوتاهی حدود 1 تا 3 دقیقه با حداکثر شدت و سرعت انجام می‌دهند بیشتر مشهود است. مواد و روش‌ها: دراین مطالعه از بین دانشجویان دختر ورزشکار 30 نفر از طریق آزمون7 مرحله‌ای بروس انتخاب شدند و با استفاده از روش نمونه‌گیری تصادفی به دو گروه 15 نفره تقسیم گردیدند. برای این منظور هر یک از آزمودنی‌ها که توانسته‌اند 6 مرحله از آزمون 7 مرحله‌ای را با موفقیت پشت سر بگذارند، انتخاب شدند، گروه اول برای مصرف مکمل کراتین، گروه دوم برای مصرف دارونما در نظر گرفته شدند. پیش از شروع برنامه تمرین از کلیه آزمودنی‌ها پیش‌آزمون به‌عمل آمد. بعد از انجام آزمون خون‌گیری از آزمودنی‌ها خواسته شد که به مدت یک هفته مکمل کراتین را مصرف نمایند و گروه دارونما در این مدت پودر نشاسته مصرف نمودند پس از این مدت هردو گروه تحت آزمون کانینگهام قرار گرفتند و 24 ساعت پس از آزمون از آنها مجددًا خون‌گیری به‌عمل آمد و شاخص‌های آسیب سلولی به‌وسیله کیت مخصوص اندازه‌گیری شد. مقایسه‌های آماری در محیط نرم‌افزارSPSS انجام گردید. نتایج: یافته‌های حاصل از آزمون تی مستقل تفاوت معنی‌داری را در دلتای کراتین کیناز بین دو گروه نشان نداد (183/0=P). به عبارتی، مکمل‌سازی کراتین سطوح کراتین کیناز را نسبت‌به گروه کنترل دستخوش تغییر معنی‌داری نمی‌کند. همچنین یافته‌های حاصل از آزمون تی مستقل تفاوت معنی‌داری را در دلتای لاکتات دهیدروژناز بین دو گروه نشان نداد (227/0=P). به عبارتی، مکمل‌سازی کراتین سطوح لاکتات دهیدروژناز را نسبت‌به گروه کنترل دستخوش تغییر معنی‌داری نمی‌کند. نتیجه‌گیری: نتایج این تحقیق نشان می‌دهد که یک دوره مکمل‌گیری کراتین تغییرات معنی‌داری بر سطوح کراتین کیناز و سطوح لاکتات دهیدروژناز نسبت‌به گروه کنترل ندارد و نیاز به مطالعات بیشتر است.

Title: اثر چهار هفته فعالیت هوازی در آب بر علایم بالینی و سایتوکاین‌های TNF-α و IL-10 در مدل حیوانی مالتیپل اسکلروزیس

http://knh.shmu.ac.ir/index.php/site/article/view/1818

مقدمه: با‌توجه به اهميت درمان‌هاي تکميلي، در اين پژوهش هدف بررسي اثربخشي 4 هفته تمرين هوازي شنا بر روي علایم بالینی و سایتوکاین‌های Tumor necrosis factor alpha) TNF-α) و (Interleukin 10 IL-10) در سطوح سرمی موش‌های مبتلا‌به مالتیپل اسکلروزیس از طریق القای انسفالومیلیت خود ایمن تجربی (Experimental autoimmune encephalomyelitis یا EAE) است. مواد و روش‌ها: 80 سر موش سوری ماده با نژاد C57BL/6 با سن 12-10 هفته و وزن 2±20 به 8 گروه 10 تایی (سالم کنترل، سالم شنا، MS کنترل، MS شنا، MS اینترفرون، MS اینترفرون و شنا، MS شاهد تزریق، MS شاهد شنا و تزریق) تقسیم شدند. از روز نهم بیماری، موش‌ها به مدت چهار هفته مقدار 150 IU/g اینترفرون بتا-1 دریافت و یا چهار هفته ورزش شنا روزانه نیم ساعت مواجهه داده شدند. وزن و علایم بالینی به‌صورت روزانه با ثبت امتیازات بین گروه‌ها ارزیابی می‌شد. در پایان پروتکل نمونه‌های خونی از قلب استخراج و شاخص‌ها به‌روش الایزا سنجیده شدند. نتايج: براساس نمودار امتیازات علایم بالینی، بین گروه‌های MS شنا و MS اینترفرون و MS شنا اینترفرون با گروه MS اختلاف با سطح معنی‌داری 001/0 مشاهده شد، شدت آسیب در گروه‌های MS نسبت‌به فعال به‌صورت میانگین روی امتیاز 2 تا 3 قرار گرفت و حاکی از تخفیف پیشرفت ضایعات دمیلینه حاصل از اثر ورزش در آب بوده است. نتیجه‌گیری: یافته‌ها پیشنهاد می‌دهند احتمالاً شنا نسبت‌به داروی اینترفرون بتا-1، عاملی مؤثرتر در تغییر سطح سایتوکاین‌های سرمی و بهبود علایم بالینی موش‌های مدل EAE می‌باشد. بنابراین می‌توان نتیجه گرفت که تمرین منظم ورزشی در کنار درمان‌های پزشکی می‌تواند برای کاهش اختلالات MS مفید باشد.

Title: بررسی تأثیر ماساژ اختصاصی پا بر شدت درد بیماران بعد از عمل هیسترکتومی

http://knh.shmu.ac.ir/index.php/site/article/view/1698

مقدمه: از مشکلات عمده بیماران پس از عمل هیسترکتومی درد می‌‌باشد که روند بهبودی و حال عمومی بیمار را به شدت تحت تأثیر قرار می‌‌دهد. لذا این مطالعه با هدف تعیین تأثیر ماساژ اختصاصی پا بر درد بیماران بعد از عمل هیسترکتومی انجام گردید. مواد و روش‌ها: این پژوهش یک مطالعه کارآزمایی بالینی دو گروهی است. تعداد نمونه‌ها 60 بیمار هیسترکتومی شده واجدشرایط با نمونه‌گیری در دسترس از 2 بیمارستان شریعتی و ولیعصر تهران در سال 1391 (30 مداخله، 30 کنترل) با تخصیص تصادفی و استفاده از کارت رنگی قرعه‌کشی انتخاب شدند. در گروه مداخله ماساژ پا به مدت 20 دقیقه دو مرتبه، یک بار در روز در طی دو روز متوالی بعد عمل انجام گرفت. گروه کنترل مراقبت‌های روتین را دریافت کردند. قبل و 30 دقیقه بعد از مداخله در هر دو گروه شدت درد با استفاده از مقیاس استاندارد دیداری درد (VAS) اندازه‌گیری شد. داده‌های این پژوهش با آزمون‌های تی مستقل، کای اسکوئر و تست دقیق فیشر با استفاده از نرم‌افزار آماری تجزیه و تحلیل شد (05/0<P). نتایج: نشان داد که اختلاف میانگین نمره درد قبل و بعد از دو جلسه ماساژ در گروه مداخله 56/2 و در گروه کنترل 01/1 می‌‌باشد. در نتیجه مقایسه دو گروه مداخله و کنترل از نظر کاهش شدت درد 30 دقیقه بعد از دو نوبت ماساژ اختلاف آماری معنی‌داری نشان داد (001/0P<) که می‌‌تواند ناشی از تأثیر خالص ماساژ پا باشد. نتيجه‌گيري: ماساژ اختصاصی پا می‌‌تواند به‌عنوان یک مداخله مؤثر پرستاری در کنترل درد پس از عمل هیسترکتومی مفید واقع شود.

Title: اعتباریابی نسخه فارسی مقیاس خودكارآمدي استفاده مناسب از دارو (SEAMS) در سالمندان

http://knh.shmu.ac.ir/index.php/site/article/view/1849

مقدمه: اغلب سالمندان مبتلا‌به بيماري‌هاي مزمن همزمان چند دارو مصرف مي‌كنند و درنتیجه پایبندی به مصرف دارو در اين افراد حائز اهمیت است. این مطالعه با هدف اعتباریابی نسخه فارسی مقیاس خودكارآمدي استفاده مناسب از دارو (SEAMS: Self-efficacy for appropriate medication use scale) در سالمندان طراحي گرديد. مواد و روش‌ها: در اين مطالعه روش‌شناختي 30 سالمند 60 سال و بالاتر واجدشرايط ورود به مطالعه، به‌صورت نمونه‌گیری تصادفی از مراکز بهداشتی درمانی گنبد كاووس و براساس کد پرونده پزشکی انتخاب شدند. اطلاعات دموگرافیک، پرسشنامه MMSE (The mini–mental state examination) و همچنین پرسشنامه SEAMS برای شرکت‌کنندگان تکمیل گردید. پس از تعیین روایی صوری و محتوایی پرسشنامه، ثبات داخلی و پایايی ابزارهای مورد پژوهش از طریق آلفای کرونباخ، روش دو نیمه کردن و آزمون مجدد با استفاده از نرم‌افزار SPSS مورد بررسی قرار گرفت. نتایج: میانگین سن شرکت‌کنندگان 9/67 سال بود. روایی صوری به‌روش کیفی (مصاحبه با 10 نفر سالمند گروه هدف) و روایی محتوا (پنل متخصصان) بررسی و تأیید شد. پایایی درونی با استفاده از آلفای کرونباخ 81/0 و با استفاده از روش دو نیمه کردن 77/0 به‌دست آمد. همچنین پایايی بیرونی ابزار با استفاده از آزمون مجدد مورد ارزیابی قرار گرفت و ضریب همبستگی اسپیرمن حاصل 97/0 بود. نتیجه‌گیری: با‌توجه به نتايج اين مطالعه مقیاس SEAMS از روایی و پایایی مناسبي در میان سالمندان ایرانی برخوردار است لذا براي ارزيابي پايبندي به درمان در سالمندان مبتلا‌به بيماري‌هاي مزمن توصيه مي‌شود.

Title: ارزیابی کارایی بیمارستان‌های استان سمنان با استفاده از تحلیل پوششی داده‌ها با ماهیت ورودی

http://knh.shmu.ac.ir/index.php/site/article/view/1853

مقدمه: بیمارستان‌ها به‌عنوان یکی از مراکزی که بیشترین منابع نظام سلامت را مصرف می‌نمایند بر حسب نوع فعالیت، دارای عملکرد و کارایی متفاوتی هستند. هدف از انجام این مطالعه، ارزیابی کارایی بیمارستان‌های استان سمنان براساس روش تحلیل پوششی داده‌ها با ماهیت ورودی می‌باشد. مواد و روش‌ها: پژوهش حاضر از نظر هدف، کاربردی و از نظر ماهیت، پیمایشی و توصیفی است و از تکنیک تحلیل پوششی داده‌ها، فرم پوششی و با ماهیت ورودی با فرض بازده متغیر نسبت‌به مقیاس، استفاده شد. در این مطالعه کارایی نسبی 13 بیمارستان استان سمنان طی سال‌های 1393 تا 1395 مورد ارزیابی قرار گرفت. اطلاعات موردنیاز از مستندات علمی و داده‌های مربوط‌به شاخص‌های ورودی و خروجی از معاونت توسعه مدیریت و برنامه‌ریزی منابع دانشگاه علوم پزشکی سمنان، شاهرود و معاونت درمان تأمین اجتماعی جمع‌آوری و از نرم‌افزار GAMS برای تحلیل داده‌ها استفاده شد. نتایج: میانگین کارایی بیمارستان‌ها، طی سال‌های 1393 تا 1395 به‌ترتیب 76/0، 82/0 و 81/0 بوده و همچنین 38/0 درصد بیمارستان‌ها از بازدهی به مقیاس ثابت، 38/0 درصد از بازدهی به مقیاس کاهشی و 24/0 درصد از بازدهی به مقیاس افزایشی برخوردار بودند، بنابراین 76/0 درصد بیمارستان‌های مورد بررسی، قابلیت توسعه بیش از میزان فعلی را ندارند. نتیجه‌گیری: اغلب بیمارستان‌ها به‌صورت کارا عمل نمی‌کنند. بیمارستان‌های ناکارا می‌توانند با الگوگیری از بیمارستان‌هایی که به‌عنوان واحد مرجع شناخته شده‌اند و با مدیریت بهتر منابع مالی و انسانی، به مرز کارایی نزدیک شوند. اجرای نتایج این تحقیق، می‌تواند ضمن معرفی الگوهای عملکردی به مدیران عالی بخش سلامت، امکان برنامه‌ریزی دقیق‌تر برای توسعه ظرفیت خدمات بهداشتی درمانی و صرفه‌جویی در منابع را فراهم کند.
